# Low Functional *β*-Diversity Despite High Taxonomic *β*-Diversity among Tropical Estuarine Fish Communities

**DOI:** 10.1371/journal.pone.0040679

**Published:** 2012-07-09

**Authors:** Sébastien Villéger, Julia Ramos Miranda, Domingo Flores Hernandez, David Mouillot

**Affiliations:** 1 Laboratoire Évolution et Diversité Biologique, Université Paul Sabatier, Toulouse, France; 2 Centro de Ecología, Pesquerías y Oceanografía de Golfo de México, Universidad Autónoma de Campeche, Campeche, México; 3 ARC Centre of Excellence for Coral Reef Studies, James Cook University, Townsville, Queensland, Australia; 4 Ecologie des systèmes marins côtier, Université Montpellier 2, Montpellier, France; Michigan State University, United States of America

## Abstract

The concept of *β*-diversity, defined as dissimilarity among communities, has been widely used to investigate biodiversity patterns and community assembly rules. However, in ecosystems with high taxonomic *β*-diversity, due to marked environmental gradients, the level of functional *β*-diversity among communities is largely overlooked while it may reveal processes shaping community structure. Here, decomposing biodiversity indices into α (local) and *γ* (regional) components, we estimated taxonomic and functional *β*-diversity among tropical estuarine fish communities, through space and time. We found extremely low functional *β*-diversity values among fish communities (<1.5%) despite high dissimilarity in species composition and species dominance. Additionally, in contrast to the high α and *γ* taxonomic diversities, α and *γ* functional diversities were very close to the minimal value. These patterns were caused by two dominant functional groups which maintained a similar functional structure over space and time, despite the strong dissimilarity in taxonomic structure along environmental gradients. Our findings suggest that taxonomic and functional *β*-diversity deserve to be quantified simultaneously since these two facets can show contrasting patterns and the differences can in turn shed light on community assembly rules.

## Introduction

Partitioning biological diversity across spatial scales has been focused on in numerous ecological studies for several decades since the pioneering works of Whittaker [1], [2]. The local and regional diversities were called *α-* and *γ*-diversities, respectively, while dissimilarity between these two scales was coined as *ß-*diversity [1], [3]. *ß-*diversity is thus a key component of biodiversity since measuring whether communities share similar species is crucial for understanding the driving forces underlying community structure at multiple spatial scales [4], [5], [6] as well as for conservation purposes [7], [8].

In studies investigating diversity partitioning among sites, biodiversity indices are almost exclusively based on species composition even though the definition of biodiversity includes various facets of the diversity of life [7], [9]. Most current measures of *ß-*diversity ignore what makes communities different over space and time: species relative abundances and species biological features. Indeed, for most *ß-*diversity indices (e.g. Jaccard, Sorensen indices), the maximum value is reached when the communities have no species in common [10]. This kind of species-based approach is an incomplete view of community structure. However, rapid movement has been occurring in this field, with recent studies that start to include phylogenetic and functional differences among species when assessing dissimilarity between communities [5], [7], [11], [12], [13], [14], [15], [16]. Indeed, two communities can be very dissimilar in terms of species composition but very similar in terms of biological composition regarding trophic levels [17], morphological traits [18] or phylogenetic lineages [19]. In other words, should a set of communities with no species in common be always assigned the highest possible *ß*-diversity value? A negative answer to this question raises two often overlooked issues. What is the level of functional *ß*-diversity among communities when taxonomic *ß*-diversity is high? What can the examination of taxonomic and functional *ß*-diversity teach us about the ecological processes shaping community structure?

The potential of functional traits to reveal processes structuring communities has been recently emphasized [20], [21], [22]. More particularly, comparing taxonomic and functional beta-diversity levels can disentangle community assembly rules [13], [14], [15], [16], [23]. For instance, a strong niche filtering process along an environmental gradient will induce a high level of taxonomic dissimilarity coupled to a high level of functional dissimilarity as the dominant functional strategies will vary along the gradient [13], [14], [16]. In contrast, if neutral processes are predominant, then taxonomic and functional beta-diversity should not differ from random association between species abundance and functional traits.

Fish communities inhabiting tropical estuaries provide a unique opportunity to investigate functional diversity partitioning because (*i*) the functional traits of the fish have already revealed mechanisms underlying community structure [24], [25], (*ii*) estuarine communities are generally species-rich [26] and such communities are necessary to implement null models of community structure and (*iii*) estuaries present high variability in environmental conditions (mainly salinity) which often forces a high species turnover across space and time [17], [27]. Moreover, tropical estuarine ecosystems are of primary concern for human population welfare since they provide various services of high value (protein source, regulation of pollution, recreational areas) although they can be severely impacted by human activities [28]. Fishes (teleosts and chondrichthyes) constitute a key component of biodiversity in estuaries since they have a large range of morphologies, life-history traits, behaviors, and diets, and thus are central in controlling fluxes of matter and energy within aquatic systems [29].

To contribute to the estimation of functional *ß*-diversity among communities, a large dataset of estuarine fish communities was collected and a set of functional traits related to fish diet and fish locomotion was measured. Then, we assessed the *α, γ* and *ß* components of taxonomic and functional diversity of fish communities using a common framework based on the concept of equivalent number of species [12], [30]. Since estuarine ecosystems experience high variability in environmental conditions across space and time [17], [31] we investigated taxonomic and functional diversity patterns spatially and temporally. We further tested whether the level of functional *ß*-diversity observed among communities is different from expectations given a random association between functional traits and abundances. Finally, we highlighted the role of some functional groups that stabilize the functional structure of fish communities across space and time despite high species turnover.

## Material and Methods

### Ethics Statement

The field sampling protocol received the agreement of the Mexican National Commission for Aquaculture and Fisheries (permit number: 070503-613-03).

### The Study System

**Figure 1:**
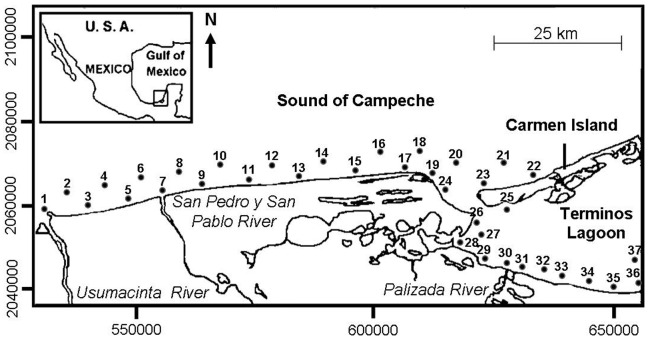
Location of the study area and of the 37 sampled stations (UTM coordinates).

The study area was located in the south of the Gulf of Mexico along the coast of the Campeche State (Mexico) (Figure 1). More precisely, the survey focused on a 150-km long transect (18°37′16N–92°42′28W to 18°30′20N–91°28′03W) of 37 stations distributed in the south-western part of the Terminos Lagoon and along the adjacent coast [17]. This transect crossed the discharge of three main rivers (the Usumacinta, San Pedro y San Pablo, and Palizada rivers) and the Carmen inlet, i.e. the exit of the Terminos Lagoon flow [32]. Local environmental conditions were highly variable through space and time (Table S1). For example, salinity ranged from 0 to 42 psu (practical salinity unit), depth from 0.8 to 12 meters and transparency from 0 to 100% of the water column depth [17]. Tide has very low amplitude (0.3 m), and thus does not significantly affect this ecosystem [32].

### Sampling Protocol

Two sampling campaigns were conducted, one from February 2003 to January 2004 and the other from February 2006 to January 2007. No major environmental changes were noticed between the two campaigns which were used separately to reinforce the robustness of our findings. Each campaign consisted of a monthly biological survey of the 37 stations (Figure 1) localized using a Global Positioning System with a precision of 75 m. Fish communities were sampled using a shrimp-trawl (length: 5 m, mouth opening diameter: 2.5 m, mesh size: 19 mm) towed for 12 minutes at a constant speed of 2.5 knots. The volume sampled was thus of 4,500 m^3^. This active sampling method is well suited to fishes living in this shallow coastal area since they are relatively small (juveniles or sub-adults and adults of standard length<30 cm) and slow swimmers. For each sample, all individuals were identified at the species level and weighed to the nearest decigram.

### Morphological Traits

For several decades, many studies have focused on the assessment of fish ecological niches through morphological traits (e.g. [25], [33], [34]). During the 2006–07 sampling campaign, a maximum of 20 individuals per species were randomly selected. On each of these individuals, 16 morphological traits were measured to describe fish functional niche. See Text S1, Figure S1 and Table S2 in Supporting Information for more details about trait assessment. This set of traits aims to quantify, as well as possible, two key functions performed by fish: food acquisition and locomotion [35]. The correlations between the 16 traits were globally weak (mean ±sd absolute value of Pearson coefficient 0.26±0.20) which illustrate their complementarity (see Table S3).

For each species, mean trait values were finally computed from individual measures assuming that intraspecific variations were lower than interspecific variations [36].

In particular, ontogenic changes were not considered as the studied species were mainly represented by juveniles and sub-adults, thus exhibiting a relatively small size-range.

Then, for each trait, mean values were standardized so that the mean was 0 and standard deviation was 1. Functional distances among fish species pairs were estimated using the Euclidean distance on standardized functional trait values. This raw functional distance matrix was then standardized by dividing it by its maximal value to obtain the operational distance matrix 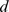 [12].

### Partitioning Functional Diversity into *α*, *β* and *γ* Components

We studied functional *ß*-diversity among fish communities belonging to the same stratum. These strata were defined both through time (between months for a given site) and space (between sites for a given month). Thus, for each of the two periods (2003–2004 and 2006–2007), sampling points were grouped into 12 temporal strata (37 stations for each month) and 37 spatial strata (12 months for each station). Samples with no fish were removed prior to statistical analyses, thus some strata actually contain less than 12 months or less than 37 sites. The two periods were used as replicates to strengthen our conclusions.

Functional *ß*-diversity was estimated using the decomposition of Rao’s quadratic entropy index [12], [37]. Let us consider *N* local communities with a global species richness of *S_G_*. Each local community *k* has a species richness *S_k_*. Abundance (here biomass) of species *j* in community *k* is noted *A_kj_*. Relative abundance of species *j* in community *k*, noted *p_kj_*, is computed as: 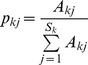, thus 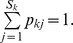


Our sampling protocol provides robust differences in local abundances, thus the relative abundance of species *j* at the stratum scale noted *p_.j_*, has to be computed as: 
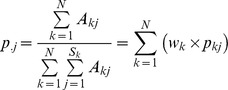
 with *w_k_* being the weight of community *k*:
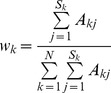
 [38].

For a community with *S* species, Rao’s quadratic entropy 
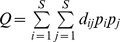
is maximal when two conditions are met: all species present are maximally dissimilar and have equal abundances, i.e. for all *i* = 1,2,…,*S* 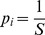 and for all *i*≠j 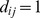 (while as usual for all *i* = j 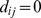). In this case, maximal quadratic entropy is 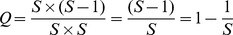.

Following its definition, the equivalent number of species 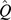 is the number of maximally dissimilar species having equal abundance which produces maximal entropy. Thus, by replacing *S* by 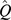 in the previous equation, we obtain 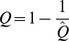 and 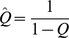 [12].

Each local diversity 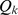 is computed following species local relative abundances and pairwise functional distances using the classical quadratic entropy formula: 
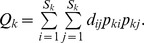


Then the mean local diversity 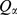, is computed as 
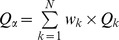
 where the weights of the communities *w_k_* are the same as those used for the computation of relative abundances at the stratum scale to ensure Q concavity [12], [37].

Finally, mean local diversity is transformed to its equivalent number of species 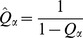 [12].

Similarly, the equivalent number of species for stratum diversity 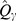 is computed based on species relative abundances at the stratum scale as:
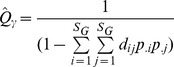



Following the multiplicative framework built on the equivalent number of species, functional *ß*-diversity (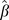) is then the ratio between global and local diversity: 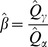 [30], [37].

By definition, functional *ß*-diversity is minimal when all the local communities have identical functional structure. In this extreme case, the functional structure of the stratum is the same as that of local communities and thus average local diversity equals regional diversity, i.e. 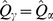 and consequently 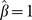. In contrast, when functional structures of local communities strongly diverge 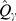 becomes higher than 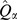 and then 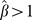. More precisely, Riccotta and Szeidl [12] demonstrated that the 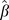 index follows the replication principle, which postulates that “when *N* equally diverse, equally large, and maximally dissimilar assemblages are pooled, the diversity of the pooled assemblage must be *N* times the diversity of the individual assemblages, i.e. 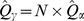
”. In other words, the maximal possible value of the functional *ß*-diversity index equals the number of communities considered (*N*). Consequently, to have an index allowing comparison of functional *ß*-diversity values between study cases having different numbers of local communities, we propose a standardized measure which ranges between 0 and 1: 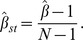


Functional *ß*-diversity based on species abundances and functional distances (hereafter noted *ß_FA_*) was computed for each temporal and spatial stratum using the formula detailed above. We also measured functional *ß*-diversity based only on functional composition (hereafter noted *ß_FC_*). To this aim, for each local community, the abundance of each of the *k* species present was set to 1/S_k_, which guarantees that all the local communities have the same weight for a given stratum. In other words, *ß_FC_* differs from *ß_FA_* by considering equal contribution of species to local diversity and equal contribution of local communities to the stratum diversity.

### Measuring Taxonomic *β*-diversity

We measured taxonomic *ß*-diversity in two ways in order to determine the relative effects of species identities and species abundances on the level of *ß*-diversity observed among fish communities.

First, we quantified the taxonomic *ß*-diversity including species abundances (hereafter noted *ß_TA_*) using the framework proposed by Jost [30]. This taxonomic *ß*-diversity index is based on the concept of equivalent number of species applied to the Shannon entropy. More specifically, Jost’s measure of relative homogeneity was converted to a relative heterogeneity measure to be analogous to *β*-diversity (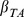), 
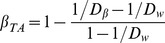
, with 
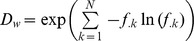
 and 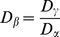, where 
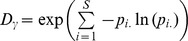
 and 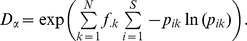



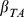
equals 0 when communities share the same species and when species have the same relative abundances in all communities. It equals unity when communities have no species in common whatever species abundances.

We also measured taxonomic *ß*-diversity based only on species composition (hereafter noted *ß_TC_*) using the same decomposition after setting all species abundances to 1/S_k_.

We assessed the environmental variability in each stratum by computing the average Euclidean distance among communities based on standardized values of depth, transparency, salinity and dissolved oxygen. Then, we estimated the correlation between this environmental variability and the four metrics of *ß*-diversity.

### Testing Assembly Rules

Observed α, γ and *ß* values are informative *per se* to assess the diversity and level of dissimilarity in the taxonomic and functional structure of communities. To this aim, the use of taxonomic and functional *ß-*diversity** indices based on the same framework (i.e. equivalent number of species) and which have the same potential range is particularly useful.

**Figure 2:**
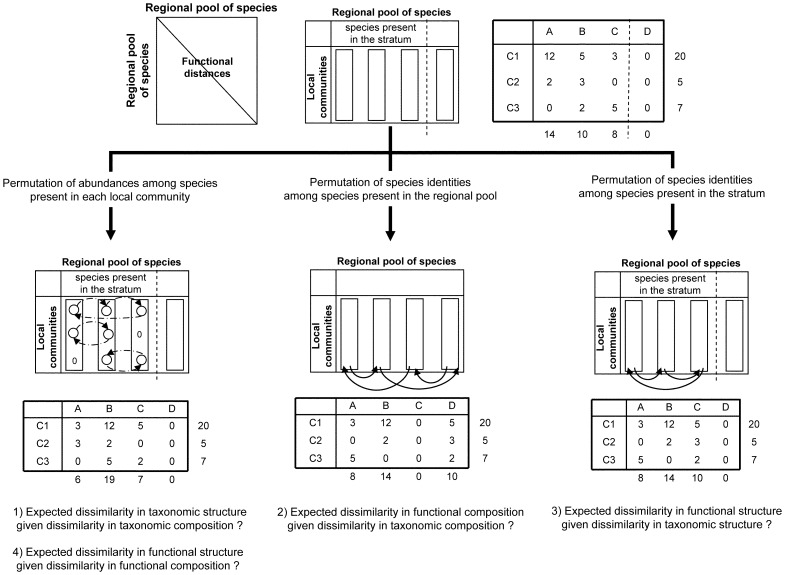
Schematic representation of the null-models used. Matrices of species abundances in set of communities (i.e. a stratum) and of pairwise functional distances are illustrated at the top. Note that species present at the stratum scale are a subset of the species present at the regional scale (i.e. global area of the study) and thus some columns of the abundance matrix contain only null values. A hypothetical example is provided on the top right, with the sum of columns (i.e. species abundances at the stratum scale) and lines (i.e. total abundances in local communities). The first procedure consists in shuffling abundances among the species present in each local community. This generates expected values of dissimilarity in taxonomic structure given the observed dissimilarity in taxonomic composition (null-model 1) as well as expected values of dissimilarity in functional structure given observed dissimilarity in functional composition (null-model 4). The second procedure randomly permutes columns of the abundance matrix at the regional scale to generate expected values of dissimilarity in functional composition without modifying the dissimilarity in taxonomic composition (null-model 2). The third procedure is similar but columns are permuted only among species present at the stratum scale so it produces the expected level of dissimilarity in functional structure without modifying dissimilarity in functional composition (null-model 3). None of the three permutation processes modifies the distribution of local abundances (i.e. contribution of the local communities to the total abundance of the stratum). For each procedure an illustration of output given the above example is provided.

However, one step further, it is necessary to assess whether the measured values of *ß*-diversity are significantly different from those expected under suitable ecological hypotheses [39]. Here, we aimed to test whether spatial and temporal functional *ß*-diversity observed among fish communities were due to random associations between functional identities and abundances. To address this issue, we generated four complementary null expectations using permutations of species abundances and functional identities (Figure 2).

First, dissimilarity in taxonomic structure is influenced by the level of dissimilarity in taxonomic composition as well as by the distribution of species abundances within and between local communities. For instance, given a high dissimilarity in taxonomic composition (*ß_TC_*), dissimilarity in taxonomic structure (*ß_TA_*) could be low if a few species are dominant in all local communities or in contrast could be high if there is a strong turnover in species dominance among local communities. Therefore, to ask whether non-random species dominance influences *ß_TA_* beyond its influence over *ß_TC_*, we generated a null expectation for *ß_TA_* that maintained the observed level of *ß_TC_*. The randomization procedure shuffles abundances among species present in each local community (Figure 2, null-model 1).

Another question is whether the diversity of functional identities present at the stratum scale constrains the dissimilarity in functional composition among local communities. For example, a low dissimilarity in functional composition (*ß_FC_*) despite a high dissimilarity in taxonomic composition (*ß_TC_*) could result from a filtering of the diverse functional strategies present at the regional scale and the presence of only few able to cope with conditions in local communities. Therefore to determine whether non-random ecological processes (e.g. niche filtering) influence *ß_FC_* beyond their influence over *ß_TC_*, we generated null expectations for *ß_FC_* that maintained the observed level of *ß_TC_* (null-model 2). The randomization procedure shuffles species functional identities among all the species present at the regional scale (Figure 2). Note that this procedure randomizes the functional identity of species but functional trait values were not permuted within species to prevent producing unrealistic trait combinations.

Furthermore, the functional structure of communities is determined by the association between species functional identities and their abundances. For example, given a high level of dissimilarity in taxonomic structure (*ß_TA_*), the dissimilarity in functional structure (*ß_FA_*) would be low if the dominant species at the stratum scale are functionally close. Therefore, we tested whether *ß_FA_* was significantly different from the null expectation postulating a random association between functional identities and species abundances at the stratum scale but keeping *ß_TA_* constant. The permutation procedure for this null model randomly shuffles functional identity among species present at the stratum scale (Figure 2, null-model 3).

Finally, to determine whether a non-random association between functional strategies at the local scale influences *ß_FA_* beyond its effect on *ß_FC_*, we generated a null expectation for *ß_FA_* that maintained the observed level of *ß_FC_* (null-model 4). The procedure used to generate this fourth null-expectation was the same as that used to generate *ß_TA_* given the observed *ß_TC_* (Figure 2). Indeed, the random permutation of abundances among species present in each local community does not modify the functional strategies present in each community while permuting the association between local abundances and functional identities.

For each null-model, 999 randomizations were carried out in each stratum. Then, observed *β*-diversity (functional or taxonomic) values were compared to the distribution of simulated *ß*-diversity values obtained under each null hypothesis to obtain p-values [40]. Thus, considering a two-sided test with a global risk of 5%, a *p*-value lower than 2.5% indicated a *β*-diversity lower than expected whereas a *p*-value higher than 97.5% indicated a *β*-diversity higher than expected [40].

### Functional Groups

To visualize the relative position of fish species in the functional space, a Principal Coordinates Analysis (PCoA) was carried out on the standardized functional distance matrix *d*. In addition, species were gathered into 20 functional groups based on their pairwise functional distances, using a “partitioning around medoids” algorithm (“pam” function in R) which searches for the clustering that minimizes the distances among species within each functional group. Species and group dominance were explored in terms of biomass and occurrence both at sample and stratum scales.

Calculation of indices and statistical analyses were carried out using R statistical software [41]. The script for computing functional *β*-diversity is provided as Text S2.

## Results

### Data Collected

A total of 46,012 and 25,639 individuals were caught in 2003–2004 and 2006–2007 respectively for corresponding weights of 557 and 398 kg. Species richness was 87 for both periods while total species richness was of 105 species over the two periods.

The 16 functional traits were measured on 1021 individuals belonging to 70 species. Among these 70 species, the 16 functional traits were estimated on 20 individuals for 40 species and on more than 10 individuals for 50 species. Indeed, some fish species were very rare and thus not captured in sufficient numbers during the 2006–07 campaign, preventing an estimation of their functional traits. Consequently, the rare species were not included in our study but their low biomass would only marginally influence the estimations of our functional diversity components that are based on abundances.

The 16 samples where the biomass of species functionally characterized represented less than 80% of community biomass were not considered in the analyses. For the remaining communities, the biomass belonging to species not functionally characterized was removed before conducting analyses.

### Observed Taxonomic and Functional Diversities

**Figure 3:**
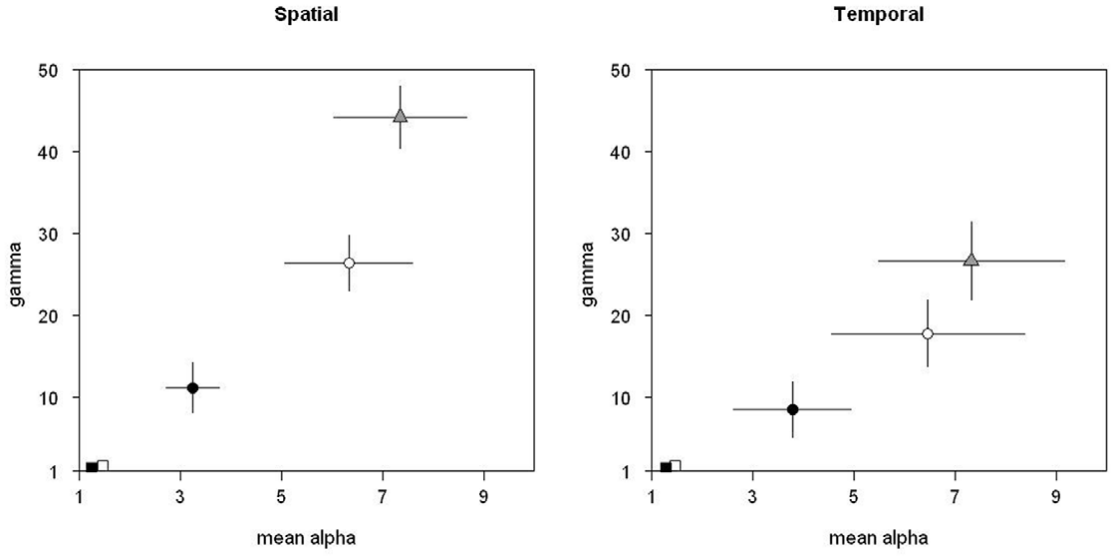
Taxonomic and functional diversity at local and regional scale. Local (mean α) and regional (γ) components are expressed as equivalent number of species (mean±SD), of taxonomic (circles) and functional (squares) diversities computed on community composition (white) or abundance structure (black) for temporal and spatial strata. The grey triangles represent species richness.

Species richness was relatively high with a mean of more than 7 species in each sample and a mean global richness at the stratum scale higher than 40 for temporal strata and higher than 25 for spatial ones (Figure 3). Taxonomic α- and γ-diversity measured using the Shannon diversity index and expressed as equivalent number of species were lower than species richness but higher than 3 and 8 for temporal and spatial strata respectively.

**Figure 4:**
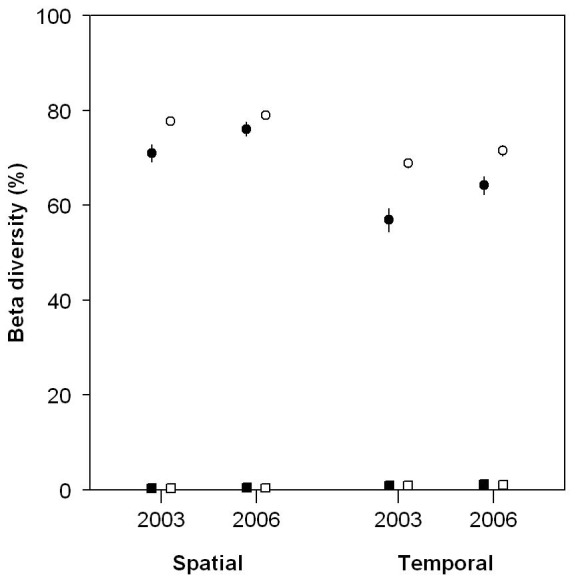
Taxonomic and functional *ß*-diversity. Taxonomic (circles) and functional (squares) *ß*-diversity values, based on community composition (white) or abundance structure (black), for the two scales of interest (mean±SE).

Taxonomic *ß-*diversity based only on species composition (*ß_TC_*) was high with values of around 75% (Figure 4). Similarly, taxonomic *ß-*diversity accounting for species abundances (*ß_TA_*) was >70% among temporal strata and >55% among spatial strata. Therefore, as expected, the taxonomic structure of fish communities in the Terminos region exhibits a strong variation both temporally and spatially.

In contrast, the two functional *ß-*diversities (based only on functional composition *ß_FC_*, and on functional structure *ß_FA_*) were both very low with values lower than 1.5% (Figure 4). These low turnover values resulted from both low α and γ-diversity, with respective equivalent number of species close to 1 (i.e. the minimum value possible).

Taxonomic *ß-*diversity based only on species composition (*ß_TC_*) was positively correlated to environmental heterogeneity within strata (Pearson’s correlation r = 0.43, p<0.001). In contrast, taxonomic *ß-*diversity accounting for species abundance *ß_TA_* was weakly related to environmental heterogeneity (r = 0.19, p = 0.06). The two metrics of functional *ß-*diversity, *ß_FC_* and *ß_FA_*_,_ were negatively (r = −0.12 and −0.14, respectively), although not significantly, related to environmental heterogeneity.

### Null Models Outputs

The first null model revealed that the observed high taxonomic *ß-*diversity (*ß_TA_*) is significantly lower than expected given the strong dissimilarity in species composition (*ß_TC_*) and the local abundance distributions among species (Table 1).

**Table 1:** Statistics obtained under the four null-models (Figure 2) for the taxonomic *ß_TA_* and functional *ß*-diversity indices (*ß_FC_* and *ß_FA_*).

	Null-model 1: Expected dissimilarity in taxonomic structure (β*_TA_*) given observed dissimilarity in taxonomic composition (β*_TC_*)			Null-model 2: Expected dissimilarity in functional composition (β*_FC_*) given observed dissimilarity in taxonomic composition (β*_TC_*)			Null-model 3: Expected dissimilarity in functional structure (ß*_FA_*) given observed dissimilarity in taxonomic structure (β*_TA_*)			Null-model 4: Expected dissimilarity in functional structure (β*_FA_*) given observed dissimilarity in functional composition (β*_FC_*)		
	−	ns	+	−	ns	+	−	ns	+	−	ns	+
Temporal 2003	100	0	0	8	92	0	8	92	0	33	67	0
Temporal 2006	83	17	0	0	100	0	0	100	0	8	92	0
Spatial 2003	68	32	0	8	92	0	3	97	0	32	68	0
Spatial 2006	46	54	0	0	100	0	0	95	5	21	76	3

Values represent the percentage of strata in which p-values indicate that the observed *ß*-diversity is significantly lower (−), not significantly (ns) different and significantly higher (+) than expected under the corresponding null hypothesis.

The results of the second null model showed that functional *ß-*diversity based only on functional composition (*ß_FC_*) is almost always not significantly different from those obtained with a random assignment of functional identities, given both the observed dissimilarity in species composition (*ß_TC_*) and the observed distribution of functional distances among species at the regional level (Table 1).

The two other null models aimed to test the determinants of the observed stability in the functional structure (i.e. low functional *ß-*diversity *ß_FA_*). First, when testing the assumption of a random association between functional identities and abundance patterns at the stratum scale (i.e. taxonomic *ß-*diversity *ß_TA_* kept constant), the observed functional *β*-diversity was significantly lower than expected in less than one fourth of the strata (Table 1). Similarly, the observed low functional *ß-*diversity was almost always not significantly different from those obtained under the null expectation suggesting a random association between species functional identities and abundance patterns at the local scale, i.e. dissimilarity in functional composition (*ß_FC_*) remained constant (Table 1).

### Functional Group Dominance

**Figure 5:**
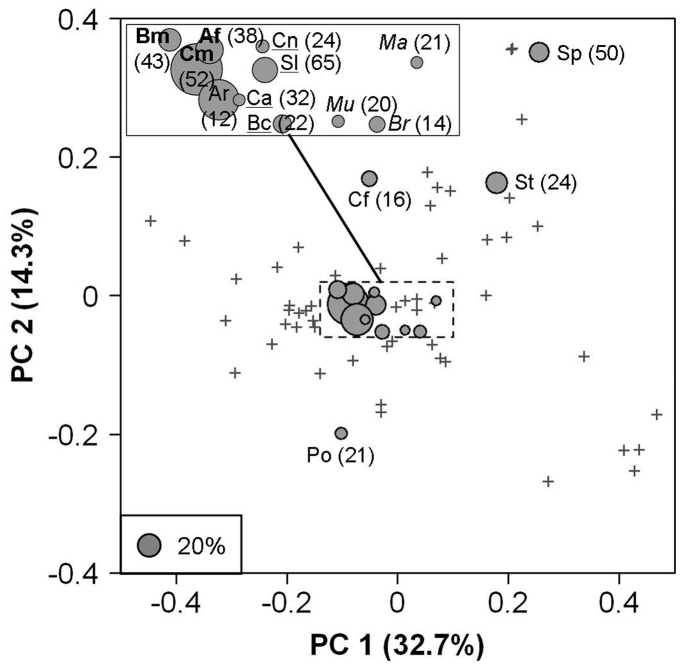
Principal Coordinates Analysis (PCoA) carried out on the 70 species using standardized functional distances. The 15 dominant species are represented by circles of sizes that are proportional to their mean relative biomass when they occur. Their percentages of occurrence over the 810 samples are given in parentheses. The other 55 species are plotted with grey crosses. Codes for dominant species names are: Af = *Ariopsis felis*, Ar = *Archosargus rhomboidalis*, Bc = *Bairdiella chrysoura*, Bm = *Bagre marinus*, Br = *Bairdiella ronchus*, Ca = *Cynoscion arenarius*, Cf = *Chaetodipteurs faber*, Cm = *Cathorops melanopus*, Cn = *Cynoscion nothus*, Ma = *Menticirrhus americanus*, Mu = *Micropogonias undulatus*, Po = *Polydactylus octonemus*, Sl = *Stellifer lanceolatus*, Sp = *Symphurus plagiusa*, St = *Sphoeroides testudineus*. Bold names are for species belonging to the sea-catfishes group (a group on Figure 6) while names underlined or in italics correspond to species from the two sciaenid groups (respectively b and d groups on Figure 6). The center part of the PCoA plane is blown up in the top left corner.

The PCoA summarizing the functional distances among species (Figure 5) clearly highlights the dominance of generalist species, i.e. species close to the centre of the functional space [42], [43]. On the contrary, the most extreme parts of the functional space contain rare specialist species, for instance six rays (right bottom corner), two carangids (left middle part) and four flatfishes (right top corner).

**Figure 6:**
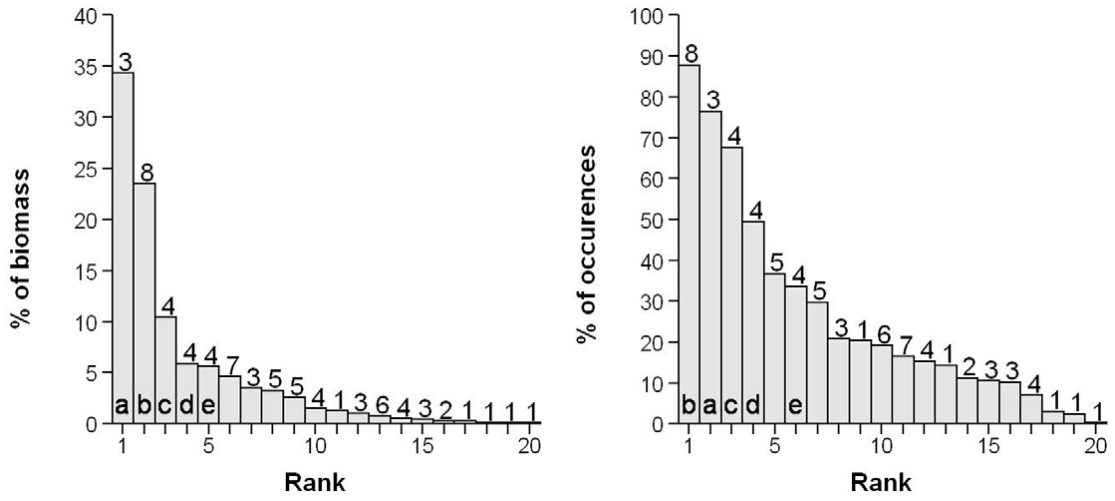
Rank-frequency diagrams representing relative abundances (left) and occurrences (right) of the 20 functional groups. The relative abundance is the mean relative biomass of the group. The percentage of occurrence is computed for each functional group over the 810 stations studied. The five most abundant groups for each function are named with letters (a-e) in the two plots. Number of species in each functional group is reported above the bars.

Species were then gathered into 20 groups based on their functional identity. The rank frequency diagrams (Figure 6) reveal a strong dominance of two groups which are frequent and abundant, the presence of an intermediate group often present but not dominant, and a right-skewed tail containing many rare groups. Among the 20 groups, the two most abundant groups contribute, on average, to 34 and 24% respectively of the total local biomass while they only contain 3 and 8 species (out of 70), respectively (Figure 6). These groups also occur very frequently (present in more than 75% of the 810 samples).

The most abundant group pools the three Ariidae sea catfish species (the dark sea catfish *Cathorops melanopus*, the hardhead *Ariopsis felis* and the gafftopsail sea catfish *Bagre marinus*). These three sea catfishes occupy the first, fourth and fifth rank (among the 70 species studied) in terms of mean relative biomass over all the samples. The second group is mainly composed of Sciaenidae species, in particular the American stardrum *Stellifer lanceolatus,* the croaker *Bairdiella chrysoura* and the two weakers *Cynoscion arenarius* and *C. nothus*, which are respectively the second, eighth, ninth and twelfth most abundant species locally. The third most abundant group pools four flatfishes which are functionally different from other fish species, including those from the two most abundant groups (Figure 5). Even though these flatfishes occurred frequently (525 occurrences over the 810 samples), they contributed only moderately to the local abundances (less than 10%).

We estimated the turnover in taxonomic structure within the two dominant functional groups in each stratum using the *ß_TA_* index [30]. The values were consistent across strata for the two functional groups with a mean of 0.32±0.16 for the sea catfish and of 0.48±0.16 for the sciaenid group, respectively. This clearly demonstrates that the dominance of these functional groups was not only determined by one ultra dominant species but, instead, by a set of species which alternatively dominate inside each group across space and time. For instance, the most abundant of the sea catfish species, the dark sea catfish *Cathorops melanopus* was present in 423 of the 810 samples whereas the sea catfish group was present in 593 samples. This species had the highest biomass in 217 samples whereas the sea catfish group was the most abundant in 349 samples. The same pattern was true for the sciaenid group which contains more species but was the dominant group in only 194 samples. Among these sciaenid species, the American stardrum *Stellifer lanceolatus* was the most widespread species (529 occurrences) but was not often the most abundant (95 samples).

## Discussion

The Terminos Lagoon area, like most estuarine ecosystems [31], is marked by a high environmental variability in terms of water salinity, depth, sediment and organic matter input [17]. We found a high fish richness in this tropical region both at regional (more than one hundred fish species) and local scales (Figure 3). This high taxonomic richness is coupled with a strong (around 65%) taxonomic *ß-*diversity, i.e. dissimilarity in taxonomic structure, both through space and time (Figure 4). However, a null-model approach revealed that the observed taxonomic *ß-*diversity is actually lower than expected given the high dissimilarity in species composition. This apparent contradiction could be explained by the dominance of few species which occur in most local communities where they furthermore contribute to most of the fish biomass. Indeed, among the 12 species present in more than one fifth of local communities, 8 contributed to more than 10% of total abundance (Figure 5).

Despite the strong taxonomic *ß-*diversity (including species abundances or not), we found extremely low functional *ß-*diversity values among fish communities (<1.5%), in terms of both functional trait composition and abundance structure. Additionally, in contrast to the high taxonomic diversity at local and stratum scales, functional diversity in local communities and in each stratum was very close to the minimal possible value. Thus, in our study case, the observed low functional *ß-*diversity results from a low functional diversity both at local (*α*) and stratum (*γ*) scales (Figure 3).

Using a null-model (Figure 2, Table 1) we demonstrate that the low dissimilarity in functional composition is not significantly different from a random expectation given the dissimilarity in species composition and the functional identities present in the regional pool of species. In other words, these results indicate that there is no strong niche filtering from the regional to the stratum scale. Species present in each stratum are thus a representative subset of the regional pool of species. As most of the species present at the regional scale are generalist species thus having similar functional identities (as illustrated on Figure 5), global functional diversity in each stratum (γ) tends to be low. Indeed, the Rao’s quadratic entropy index is maximal when all the species are equally and thus maximally dissimilar to each other [12]. In our study case most of the species were functionally close and only a few couples of them were functionally very different. This pattern is not due to the set of morphological traits we used as it was able to discriminate flatfish (e.g. “Sp” on figure 4), bentho-pelagic sea catfish (“Af”) and zooplanktonivorous pelagic species (“Po”). It rather reflects the predominance of functionally close species (sea catfish and sciaenid species). Therefore, despite the strong turnover in species composition, the species occurring in each local community are functionally close to each other leading to low local functional diversity (α). The observed low dissimilarity in functional composition thus results from the absence of niche filtering processes and the specificity of the regional pool of species which is dominated by generalist species.

The observed low functional *β*-diversity values, measured considering abundances, result from a combination of the patterns presented previously: a regional pool dominated by generalist species and the consistent presence across space and time of a few abundant species, particularly those belonging to the sea catfish and sciaenid functional groups (Figure 5, Figure 6). Indeed, these generalist species are functionally relatively close and at the same time abundant in most local communities, hence they constrain α-functional diversity to be low. Furthermore, these functional groups do not contain an ultra-dominant species but are on the contrary structured by several co-dominant species which replace each other through space and time, allowing the dominance of these groups whatever the environmental conditions and species composition. This statement is supported by correlation results between β-diversity components and environmental variability suggesting that dominant species are consistently present in all samples (*ß_TA_* weakly related to environmental heterogeneity within strata) while rarer species occur if environmental conditions are suitable (*ß_TC_* highly related to environmental heterogeneity). In other words generalist species in terms of functional traits (*sensu* Elton) also tend to be generalists in terms of environmental conditions (*sensu* Grinnell) stabilizing the functional structure of communities across space and time. However we recognize that embracing the Eltonian and Grinnellian niches using a set of traits remains challenging. Further investigations on community assembly rules may include traits that are closely related to species functions in the ecosystem and to species responses to various environmental stressors after performing experiments if necessary [44].

## Conclusion

Diversity partitioning among scales has been investigated for more than 40 years based on the taxonomic composition of communities [1], [2], [30], [45], but an increasing number of studies are now considering biological distances between species [5], [7], [11], [13], [16], [46]. Here, using a procedure to decompose *γ*-functional diversity into independent *α* and *ß*-components, we demonstrate that a low functional *β*-diversity occurs among tropical estuarine fish communities (temporally and spatially) despite high taxonomic *β*-diversity both in terms of species composition and community structure.

Overall, our results suggest that the low functional *ß-*diversity observed in this ecosystem is mainly attributable to the ultra dominance of a few functionally similar species. However, going one step further, it would be challenging to analyze the determinants of this low proportion of specialist species in the regional pool. This could be achieved by comparing the regional pool of species present in the Terminos lagoon region with the pool of species present at a larger spatial scale (e.g. southwestern part of the Gulf of Mexico) to test whether there is a filtering between this global pool and the regional one studied here. Indeed, it could be argued that this estuarine ecosystem is favorable to bentho-pelagic omnivorous species which can feed on a large variety of prey and move across portions of the ecosystem depending on abiotic conditions. In contrast, other functional strategies typically dominant in seagrass meadows or reefs (e.g. herbivorous sedentary species such as Tetraodontiformes) are marginal as these habitats are becoming scarce in the region studied here [35].

The functional approach, focusing on functional attributes of species rather than only on their taxonomic identity, has the conceptual advantage of providing ecological conclusions transposable to ecosystems that host different species [22]. Therefore, an important challenge is to test whether the stable functional structure found across the fish communities of Terminos lagoon still holds when considering other estuarine assemblages (tropical or temperate) or even other aquatic ecosystems with high taxonomic *ß*-diversity. Moreover, analyzing patterns of functional *ß*-diversity has the potential to provide indications on the processes that structure communities over spatial and temporal scales [47]. One step further, a challenging issue will be to link these *ß*-diversity patterns to ecosystem functioning and stability.

## Supporting Information

Figure S1 **Morphological traits.** Morphological traits measured on digital pictures (a): *Bl* body standard length, *Bd* body depth, *CPd* caudal peduncle minimal depth, *CFd* caudal fin depth, *CFs* caudal fin surface, *PFi* distance between the insertion of the pectoral fin to the top of the body, *PFb* body depth at the level of the pectoral fin insertion, *PFl* pectoral fin length, *PFs* pectoral fin surface, *Hd* head depth along the vertical axis of the eye, *Ed* eye diameter, *Eh* distance between the centre of the eye to the bottom of the head, *Mo* distance from the top of the mouth to the bottom of the head along the head depth axis; and with an electronic caliper (b) : *Bw* body width, *Md* mouth depth, *Mw* mouth width.
(JPG)

Table S1 **Environmental heterogeneity.** Values are means and standard deviations of coefficients of variation (%) in each stratum for four main environmental variables.
(DOC)

Table S2 **List of the 16 functional traits used.** Codes for morphological measures are the same as in figure S1. *GRl* is length of the longest gill raker and *Gl* is length of the gut from the oesophagus to the anus. The logarithm of the mass was also considered.
(DOC)

Table S3 **Pairwise Correlations (Pearson’s coefficient) between the 16 functional traits.** Traits codes are provided in Appendix A. Values in bold are significantly different from 0 with a p-value lower than 5%. The mean of the absolute correlations is 0.26 (sd = 0.20) and only 17 out of the 120 pairs of traits show a correlation higher than 0.5 in absolute value.
(DOC)

Text S1 **Functional characterization of fishes.**
(DOC)

Text S2 **betaQmult: R function to compute functional beta-diversity based on the multiplicative decomposition of the Rao's quadratic entropy.**
(TXT)
