# Supplementary figures and images for: Low Functional β-Diversity Despite High Taxonomic β-Diversity among Tropical Estuarine Fish Communities
Source: PLoS One. 2012 Jul 9;7(7):e40679. doi: 10.1371/journal.pone.0040679 (PMC3392234; doi:10.1371/journal.pone.0040679)

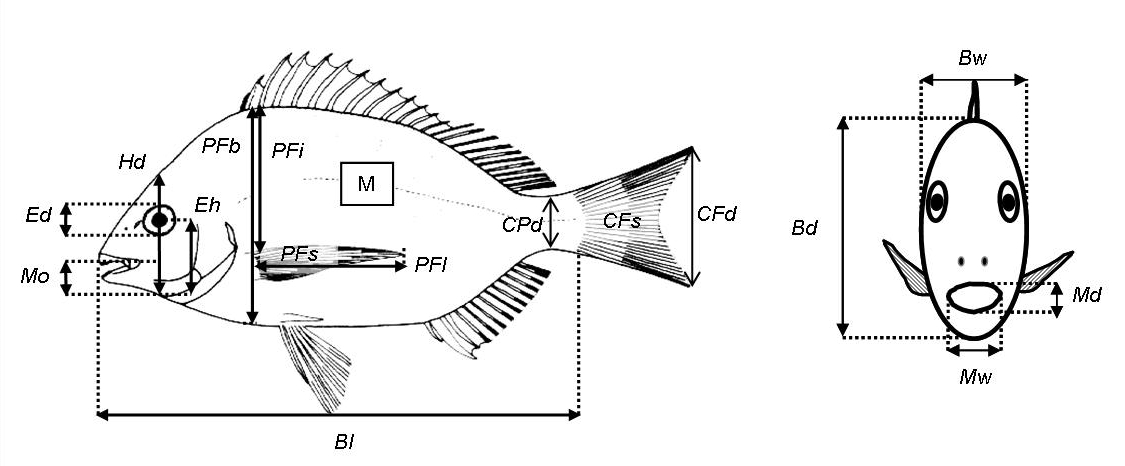

Supplement: Figure S1 — Morphological traits. Morphological traits measured on digital pictures (a): Bl body standard length, Bd body depth, CPd caudal peduncle minimal depth, CFd caudal fin depth, CFs caudal fin surface, PFi distance between the insertion of the pectoral fin to the top of the body, PFb body depth at the level of the pectoral fin insertion, PFl pectoral fin length, PFs pectoral fin surface, Hd head depth along the vertical axis of the eye, Ed eye diameter, Eh distance between the centre of the eye to the bottom of the head, Mo distance from the top of the mouth to the bottom of the head along the head depth axis; and with an electronic caliper (b) : Bw body width, Md mouth depth, Mw mouth width. (JPG) [file pone.0040679.s001.jpg]
