# Supplementary material for: Low Functional β-Diversity Despite High Taxonomic β-Diversity among Tropical Estuarine Fish Communities
Source: PLoS One. 2012 Jul 9;7(7):e40679. doi: 10.1371/journal.pone.0040679 (PMC3392234; doi:10.1371/journal.pone.0040679)
Supplement: Table S1 — Environmental heterogeneity. Values are means and standard deviations of coefficients of variation (%) in each stratum for four main environmental variables. (DOC) [file pone.0040679.s002.doc]

**Supporting Information**

**Low functional *β*-diversity despite high taxonomic *β*-diversity among tropical estuarine fish communities**

Sébastien VILLÉGER^*^, Julia RAMOS MIRANDA, Domingo FLORES HERNANDEZ and David MOUILLOT

*[*sebastien.villeger@univ-tlse3.fr*](mailto:sebastien.villeger@univ-tlse3.fr)

**Table S1. Environmental heterogeneity.**

Values are means and standard deviations of coefficients of variation (%) in each stratum for four main environmental variables.

|  | Depth | Transparency | Salinity | Dissolved oxygen |
| --- | --- | --- | --- | --- |
| Temporal 2003 | 56.6 ±4.1 | 72.8 ±16.9 | 25.2 ±9.8 | 19.0 ±6.6 |
| Temporal 2006 | 55.0 ±3.7 | 56.0 ±6.8 | 29.5 ±11.2 | 24.2 ±8.1 |
| Spatial 2003 | 11.8 ±5.6 | 62.9 ±22.6 | 18.2 ±20.8 | 23.3 ±7.4 |
| Spatial 2006 | 12.3 ±5.9 | 49.0 ±12.1 | 23.1 ±24.2 | 31.5 ±7.8 |
