# Supplementary material for: Low Functional β-Diversity Despite High Taxonomic β-Diversity among Tropical Estuarine Fish Communities
Source: PLoS One. 2012 Jul 9;7(7):e40679. doi: 10.1371/journal.pone.0040679 (PMC3392234; doi:10.1371/journal.pone.0040679)
Supplement: Table S2 — List of the 16 functional traits used. Codes for morphological measures are the same as in figure S1. GRl is length of the longest gill raker and Gl is length of the gut from the oesophagus to the anus. The logarithm of the mass was also considered. (DOC) [file pone.0040679.s003.doc]

**Supporting Information**

**Low functional *β*-diversity despite high taxonomic *β*-diversity among tropical estuarine fish communities**

Sébastien VILLÉGER^*^, Julia RAMOS MIRANDA, Domingo FLORES HERNANDEZ and David MOUILLOT

*[*sebastien.villeger@univ-tlse3.fr*](mailto:sebastien.villeger@univ-tlse3.fr)

**Table S2**

List of the 16 functional traits used. Codes for morphological measures are the same as in figure S1. *GRl* is length of the longest gill raker and Gl is length of the gut from the oesophagus to the anus. The logarithm of the mass was also considered.

| **Functional trait** | **Code** | **Formula** | **Ecological meaning** |
| --- | --- | --- | --- |
| Mass | logM | *log (Mass+1)* | Size, metabolism |
| Oral gape surface | OgSf | $\frac{Mw\times Md}{Bw\times Bd}$ | Nature/Size of food items captured  (adapted from [1]) |
| Oral gape shape | OgSh | $\frac{Md}{Mw}$ | Method to capture food items [1] |
| Oral gape position | OgPo | $\frac{Mo}{Hd}$ | Feeding method in the water column  (adapted from [2]) |
| Gill raker length | GrLg | $\frac{GRl}{Hd}$ | Filtering ability or gill protection  (adapted from [2]) |
| Gut length | GtLg | $\frac{Gl}{Bl}$ | Processing of energy poor resources such as vegetation and detritus 3] |
| Eye size | EySz | $\frac{Ed}{Hd}$ | Prey detection  (adapted from [4]) |
| Eye position | EyPo | $\frac{Eh}{Hd}$ | Vertical position in the water column [5] |
| Body transversal shape | BdSh | $\frac{Bd}{Bw}$ | Vertical position in the water column and hydrodynamism [2] |
| Body transversal surface | BdSf | $\frac{\ln\left(\left(\frac{\pi}{4}\times Bw\times Bd\right)+1\right)}{\ln\left(Mass+1\right)}$ | Mass distribution along the body for hydrodynamism [6] |
| Pectoral fin position | PfPo | $\frac{PFi}{PFb}$ | Pectoral fin use for maneuverability [7] |
| Aspect ratio of the pectoral fin | PfSh | $\frac{PFl^{2}}{PFs}$ | Pectoral fin use for propulsion  (adapted from [8]) |
| Caudal peduncle throttling | CpHt | $\frac{CFd}{CPd}$ | Caudal propulsion efficiency through reduction of drag [9] |
| Aspect ratio of the caudal fin | CfSh | $\frac{CFd^{2}}{CFs}$ | Caudal fin use for propulsion and/or direction [9] |
| Fins surface ratio | FsRt | $\frac{2\times PFs}{CFs}$ | Main type of propulsion between caudal and pectoral fins [6] |
| Fins surface to body size ratio | FsSf | $\frac{\left(2\times PFs\right)+CFs}{\pi/4\times Bw\times Bd}$ | Acceleration and/or manoeuvrability efficiency [6] |

**References**

1. Karpouzi VS, Stergiou KI (2003) The relationships between mouth size and shape and body length for 18 species of marine fishes and their trophic implications. Journal of Fish Biology 62:1353-1365

2. Sibbing, FA, Nagelkerke LAJ (2001) Resource partitioning by Lake Tana barbs predicted from fish morphometrics and prey characteristics. Reviews in Fish Biology and Fisheries 10:393-437

3. Kramer DL, Bryant MJ (1995) Intestine length in the fishes of a tropical stream. 2. Relationships to diet: The long and short of a convoluted issue. Environmental Biology of Fishes 42:129-141

4. Boyle KS, Horn MH (2006) Comparison of feeding guild structure and ecomorphology of intertidal fish assemblages from central California and central Chile. Marine Ecology-Progress Series 319:65-84

5. Gatz AJ (1979) Community organization in fishes as indicated by morphological features. Ecology 60:711-718

6. Villéger S, Ramos-Miranda J, Flores-Hernandez D, Mouillot D (2010) Contrasting changes in taxonomic vs. functional diversity of tropical fish communities after habitat degradation. Ecological Applications 20:1512–1522

7. Dumay O, Tari PS, Tomasini JA, Mouillot D (2004) Functional groups of lagoon fish species in Languedoc Roussillon, southern France. Journal of Fish Biology 64:970-983

8. Fulton CJ, Bellwood DR, Wainwright PC (2001) The relationship between swimming ability and habitat use in wrasses (Labridae). Marine Biology 139:25-33

9. Webb PW (1984) Form and function in fish swimming. Scientific American 251:72-82
