# Supplementary material for: Low Functional β-Diversity Despite High Taxonomic β-Diversity among Tropical Estuarine Fish Communities
Source: PLoS One. 2012 Jul 9;7(7):e40679. doi: 10.1371/journal.pone.0040679 (PMC3392234; doi:10.1371/journal.pone.0040679)
Supplement: Table S3 — Pairwise Correlations (Pearson’s coefficient) between the 16 functional traits. Traits codes are provided in Appendix A. Values in bold are significantly different from 0 with a p-value lower than 5%. The mean of the absolute correlations is 0.26 (sd = 0.20) and only 17 out of the 120 pairs of traits show a correlation higher than 0.5 in absolute value. (DOC) [file pone.0040679.s004.doc]

**Supporting Information**

**Low functional *β*-diversity despite high taxonomic *β*-diversity among tropical estuarine fish communities**

Sébastien VILLÉGER^*^, Julia RAMOS MIRANDA, Domingo FLORES HERNANDEZ and David MOUILLOT

*[*sebastien.villeger@univ-tlse3.fr*](mailto:sebastien.villeger@univ-tlse3.fr)

**Table S3.** Pairwise Correlations (Pearson’s coefficient) between the 16 functional traits. Traits codes are provided in Appendix A. Values in bold are significantly different from 0 with a p-value lower than 5%.

The mean of the absolute correlations is 0.26 (sd = 0.20) and only 17 out of the 120 pairs of traits show a correlation higher than 0.5 in absolute value.

|  | logM | OgSf | OgSh | OgPo | GrLg | GtLg | EySz | EyPo | BdSh | BdSf | PfPo | PfSh | CpHt | CfSh | FsRt |
| --- | --- | --- | --- | --- | --- | --- | --- | --- | --- | --- | --- | --- | --- | --- | --- |
| OgSf | -0.13 |  |  |  |  |  |  |  |  |  |  |  |  |  |  |
| OgSh | **-0.56** | 0.18 |  |  |  |  |  |  |  |  |  |  |  |  |  |
| OgPo | **-0.40** | 0.10 | **0.29** |  |  |  |  |  |  |  |  |  |  |  |  |
| GrLg | 0.07 | 0.04 | 0.09 | -0.23 |  |  |  |  |  |  |  |  |  |  |  |
| GtLg | -0.23 | **0.43** | **0.41** | 0.07 | **0.26** |  |  |  |  |  |  |  |  |  |  |
| EySz | 0.08 | **-0.29** | -0.13 | -0.01 | **-0.26** | 0.01 |  |  |  |  |  |  |  |  |  |
| EyPo | **0.24** | 0.01 | **-0.49** | 0.03 | 0.00 | **-0.38** | -0.14 |  |  |  |  |  |  |  |  |
| BdSh | **-0.40** | -0.12 | **0.76** | 0.13 | -0.13 | 0.18 | 0.02 | **-0.64** |  |  |  |  |  |  |  |
| BdSf | **-0.72** | 0.04 | **0.51** | 0.18 | -0.18 | 0.13 | -0.13 | -0.18 | **0.42** |  |  |  |  |  |  |
| PfPo | **-0.36** | 0.21 | **0.47** | 0.04 | 0.02 | **0.53** | **0.26** | **-0.75** | **0.49** | 0.16 |  |  |  |  |  |
| PfSh | -0.12 | -0.03 | **0.34** | -0.23 | **0.43** | **0.24** | -0.15 | **-0.55** | **0.47** | -0.02 | **0.45** |  |  |  |  |
| CpHt | **-0.40** | 0.01 | **0.53** | 0.05 | -0.18 | 0.17 | 0.06 | **-0.53** | **0.70** | **0.40** | **0.44** | **0.37** |  |  |  |
| CfSh | **-0.39** | -0.03 | **0.50** | 0.08 | 0.01 | **0.33** | 0.23 | **-0.67** | **0.61** | **0.27** | **0.66** | **0.42** | **0.78** |  |  |
| FsRt | 0.00 | **0.38** | -0.23 | -0.20 | -0.12 | -0.04 | -0.05 | 0.2 | -0.15 | 0.07 | -0.04 | -0.03 | 0.17 | -0.11 |  |
| FsSf | **0.36** | 0.14 | **-0.26** | **-0.59** | **0.32** | -0.05 | **-0.35** | **0.34** | **-0.29** | -0.09 | **-0.37** | 0.15 | **-0.28** | **-0.36** | 0.19 |
