# Supplementary material for: Low Functional β-Diversity Despite High Taxonomic β-Diversity among Tropical Estuarine Fish Communities
Source: PLoS One. 2012 Jul 9;7(7):e40679. doi: 10.1371/journal.pone.0040679 (PMC3392234; doi:10.1371/journal.pone.0040679)
Supplement: Text S1 — Functional characterization of fishes. (DOC) [file pone.0040679.s005.doc]

**Supporting Information**

**Low functional *β*-diversity despite high taxonomic *β*-diversity among tropical estuarine fish communities**

Sébastien VILLÉGER^*^, Julia RAMOS MIRANDA, Domingo FLORES HERNANDEZ and David MOUILLOT

*[*sebastien.villeger@univ-tlse3.fr*](mailto:sebastien.villeger@univ-tlse3.fr)

**Text S1. Functional characterization of fishes**

We evaluated functional diversity of fish communities using 16 morphological traits (Table S2). All these traits, except the log-transformed mass, are ratios of morpho-anatomical measures (17 morphological, Figure S1, and 2 anatomical measures).

**Protocol for morpho-anatomical measures**

Individual biomasses were measured using an electronic balance (precision 0.1g). Body width, mouth width and mouth depth were measured using an electronic caliper (precision of 0.1mm). The other 14 morphological traits were measured via the use of digital pictures (Canon Powershot G6, 7 million pixel resolution) using Image J software, with 0.1mm precision. The length of the longest gill raker was estimated using a stereomicroscope (precision of 0.1mm). The gut (from the oesophagus to the anus) was extracted by dissection, stretched and measured to the nearest millimeter.

**Conventions used for particular morphologies**

Our set of traits is not designed for a restricted family or morphology, so it can potentially be used for all fish species from fresh and marine waters. However, for particular morphologies (species without tail, flatfishes, rays), conventions were used for morphological measures and functional trait estimations.

For flatfishes, body depth and width, mouth depth, width and position, and eye position were measured relatively to the position of the fish in its environment; in other words, the lateralization was not considered. Additionally, as flatfishes have their two eyes on the “top” of the head, *eye size* was computed as: $\frac{2\times Ed}{Hd}$.

Flatfishes were considered without functionally pectoral fins, so *Pectoral fin position* and *Aspect ratio of the pectoral fin* were fixed to 0. Similarly, for species without caudal fin, *Aspect ratio of the caudal fin* and *Fins surface ratio* were fixed to 0.

For ray species, we used conventions to generalize the traits designed for teleosts.

*Oral gape position* was fixed to 0 for species with oral gape opening on the bottom of the body. Similarly, for species with eyes on the top of the head, *eye position* was fixed to 1.

For species with a terminal oral gape and/or lateral eyes (e.g. the cow-nosed ray *Rhinoptera bonasus*) oral gape and eye position were measured as for teleosts.

*Pectoral fins* measurements were applied considering that the length of the fin was on the axis parallel to the body axis. When caudal fin was present (e.g. Atlantic guitarfish *Rhinobatos lentiginosus*) measurements were made as for teleosts.
